# Internal Bleeding Extending to the Retroperitoneum and Right Psoas With Severe Acute Respiratory Syndrome Coronavirus 2 Infection

**DOI:** 10.7759/cureus.18477

**Published:** 2021-10-04

**Authors:** Sarah Hatahet, Magdi S Yacoub, Mina Farag, Ulviyya Gasimova, Salaheldin Elhamamsy

**Affiliations:** 1 Internal Medicine, University of Novi Sad, Novi Sad, SRB; 2 Internal Medicine, Faculty of Medicine, Cairo University, Cairo, EGY; 3 General Practice, Health Education England, Birmingham, GBR; 4 Internal Medicine, Nardone Medical Associates, Pawtucket, USA; 5 Geriatrics, Brown University, Providence, USA

**Keywords:** sars-cov-2, retroperitoneal bleed, covid-19 associated coagulopathy, jehovah's witness, complications of anticoagulation

## Abstract

Coronavirus disease 2019 (COVID-19 - severe acute respiratory syndrome coronavirus 2 {SARS-CoV-2}) infection has been associated with thromboembolic events and coagulopathy, leading to a surge in the use of anticoagulants. The dose and duration of therapy differ according to the followed protocol. Several case reports documented fatal bleeding as an adverse effect of anticoagulation.

We report a case of nearly fatal retroperitoneal bleed in an otherwise healthy 60-year-old man who developed severe COVID-19 requiring ICU stay and mechanical ventilation. The development of retroperitoneal bleed led to a 50% drop in his hemoglobin. The patient happens to be a Jehovah's Witness, and the family refused blood transfusion, which added to the complexity of the situation.

Anticoagulation is associated with a potential risk of fatal bleed in critically ill COVID-19 patients. There are different protocols of anticoagulation in the management of SARS-CoV-2. The risk of bleeding vs thrombosis should be weighed on a case-by-case basis. A high degree of suspicion, early intervention, and knowledge of alternatives to blood transfusion can improve outcomes.

## Introduction

Severe acute respiratory syndrome coronavirus 2 (SARS-CoV-2) infection has been associated with thromboembolic events and coagulopathy, leading to a surge in the use of prophylactic anticoagulants. The dose and duration of therapy differ according to the followed protocol. Several case reports documented fatal bleeding as an adverse effect of anticoagulation [1].

Many healthcare systems are using anticoagulation in protocols to manage patients with coronavirus disease 2019 (COVID-19). We present a case of a patient with acute SARS-CoV-2 infection associated with cytokine dysregulation, respiratory failure, and acute respiratory distress syndrome (ARDS), who developed a life-threatening retroperitoneal bleed in the setting of prophylactic treatment with anticoagulation. The situation was complex, as the patient was a Jehovah’s Witness (JW), and his family refused blood transfusion, which led to a very difficult discussion. The patient was closely monitored and his condition has stabilized. This report is very unique as it sheds the light on a very unusual and challenging case during the COVID-19 pandemic. 

## Case presentation

A 60-year-old man with a history of hyperlipidemia presented to the hospital on April 3, 2020, with malaise, fever, myalgia, nonproductive cough, and shortness of breath. The patient’s sister and brother had been recently hospitalized and intubated in a Boston hospital after being diagnosed with severe COVID-19. The patient frequently visited his mother who had been recovering from COVID-19. The patient was admitted initially to the medical telemetry floor, he was requiring 6 L oxygen by nasal cannula, and blood work was done (Table 1).

**Table 1:** CBC/CMP results on admission CBC: complete blood picture; CMP: comprehensive metabolic panel; AST: serum aspartate aminotransferase; LDH: lactate dehydrogenase

Parameters	Ranges
Sodium	135 (136-145mEq/L)
Calcium	8.1 (8.4-10.2 mg/dL)
Magnesium	2.5 (1.5-2 mEq/L)
Ferritin	1014 (15-200 ng/mL)
AST	65 (8-40 U/L)
LDH	336 (45-90 U/L (100-250 IU/L)
Creatine kinase	368 (25-90 U/L)
Albumin	3.1 (3.5-5.5 g/dL)
D-dimer	663.35 (0-499 ng/mLFEU)
Platelet count	139 (150-400.000/mm^3^)
Neutrophils	86.4 (54-62%)
Lymphocytes	9.4 (25-33%)
Absolute neutrophils	6.35
Absolute lymphocytes	0.69
Absolute monocytes	0.26

His CT chest showed bilateral dense peripheral opacities. The patient was started on hydroxychloroquine 400 mg twice a day on day one, followed by 200 mg twice a day for four days, azithromycin 500 mg once a day for five days, and tocilizumab 8 mg/kg single infusion on the day of admission based on COVID-19 hospital protocol at the time. Later, the same day of admission, the patient developed worsening hypoxemia, was transferred to the ICU, and intubated. 

Based on hospital protocol D-dimer was checked every other day. On April 11, 2020, the patient was started on enoxaparin sodium 1 mg/kg twice a day in the setting of elevated D-dimer. Local hospital protocol at the time recommended initiation of anticoagulation based on D-dimer results being more than 3000 ng/mL or five times the upper limit of normal. The risks and benefits of anticoagulation were discussed with the patient’s family, as the patient was intubated in the ICU and couldn't have provided consent. The patient’s D-dimer readings during the hospital stay are shown in Table 2. 

**Table 2:** D-dimer readings in a timely manner during the hospital stay

Date	Result	Reference (ng/mL FEU)
April 21, 2020	10000.00	0-499
April 19, 2020	1206.19	0-499
April 17, 2020	1912.80	0-499
April 15, 2020	2949.30	0-499
April 13, 2020	4753.32	0-499
April 11, 2020	9374.57	0-499
April 09, 2020	2629.35	0-499
April 07, 2020	1211.78	0-499
April 05, 2020	1724.93	0-499
April 03, 2020	663.35	0-499

**Figure 1:**
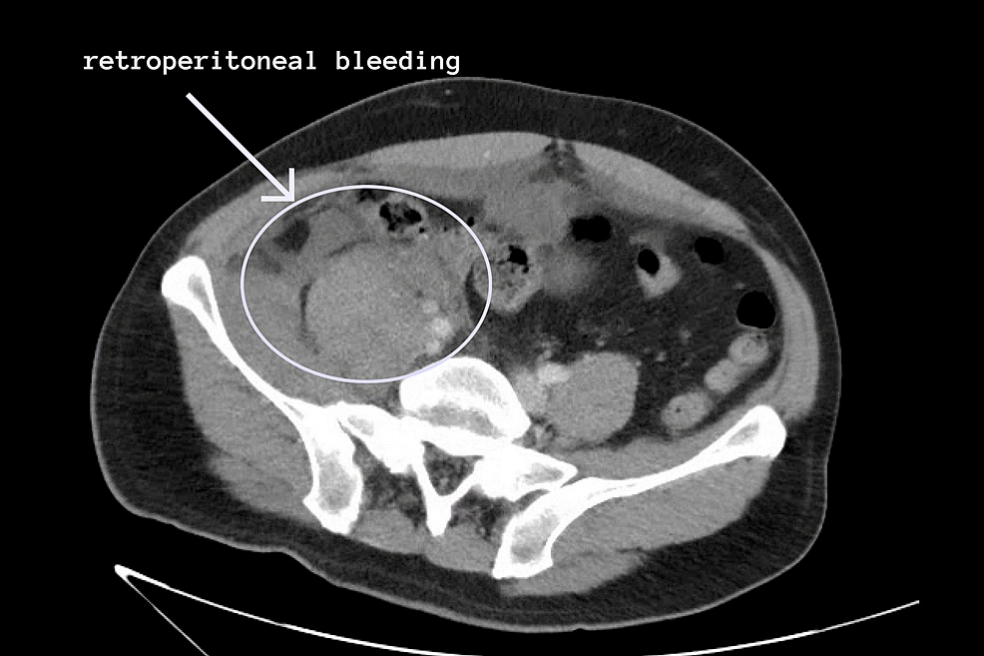
Abdominal CT with IV contrast showing acute hemorrhage in the inguinal and pelvic regions extending to the right psoas muscle and retroperitoneum.

**Figure 2:**
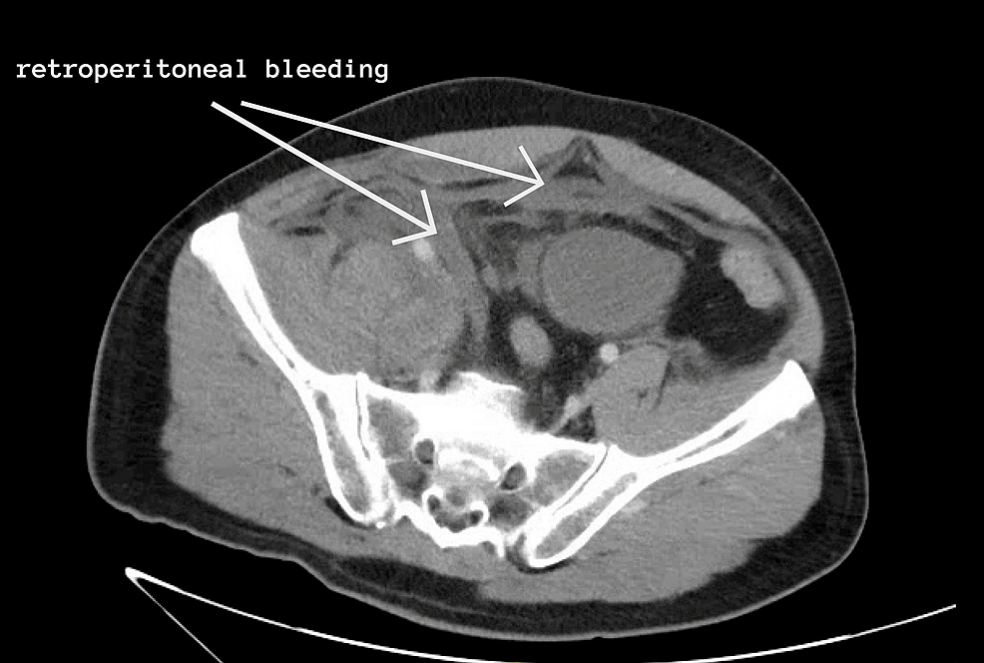
Abdominal CT with IV contrast showing acute hemorrhage in the inguinal and pelvic regions extending to the right psoas muscle and retroperitoneum.

The patient improved clinically and was extubated. However, he developed acute severe abdominal pain on April 17, 2020. The patient was investigated by CT abdomen with IV contrast, which showed acute hemorrhage in the inguinal and pelvic regions extending to the right psoas muscle and retroperitoneum (Figures 1, 2). The patient's hemoglobin dropped from 12.9 g/dL to 6.5 g/dL over the course of four days. 

The surgical team was consulted, and they recommended monitoring the hemoglobin every six hours, stopping the enoxaparin, and repeating the CT scan after 24 hours. Our team planned for interventional radiology embolization if there is evidence of active bleeding.

Enoxaparin sodium was immediately stopped and hemoglobin was closely monitored (Table 3). This was the first patient life-threatening bleed as a side effect of anticoagulation that we encountered in our hospital during the management of COVID-19. 

**Table 3:** Hemoglobin levels on anticoagulation

Date	Hemoglobin levels
April 16, 2020	12.9
April 17, 2020	10.9-9.8
April 18, 2020	9.3-7.7
April 19, 2020	7.0
April 20, 2020	6.5

That was a very challenging situation as the patient's family refused any form of blood or blood components transfusion based on the patient's beliefs as he was a JW. It was not possible to directly discuss that decision with the patient himself as he had limited insight due to toxic metabolic encephalopathy. That is why the patient was closely monitored in anticipation of potential interventional radiology, or surgical intervention. If the surgical intervention was to happen, we had the capacity of intraoperative blood salvage. 

After stopping the anticoagulation, the patient's hemoglobin stabilized at 6.5 g/dL with no further drop. Forty-eight hours later, a follow-up CT showed stabilization of the hematoma, and no surgical intervention was needed. The patient was discharged from the hospital on April 23, 2020, in stable condition. 

## Discussion

Coagulopathy is widely reported with SARS-CoV-2 infection [2]. Studies have found that COVID-19 may predispose patients to both arterial and venous thrombotic events [3]. SARS-CoV-2 infection provokes the systemic inflammatory response and causes an imbalance between procoagulant and anticoagulant homeostatic mechanisms [4]. Several pathological mechanisms were found to be associated with SARS-CoV-2 infection, such as excessive tissue inflammation, endothelial dysfunction, platelet activation, and altered hemostasis [5]. These pathological mechanisms increase the risk of developing micro- and macro-vascular thrombi, leading to high morbidity and mortality. Arachchillage and Laffan reported that abnormal coagulation parameters have been associated with poor prognosis. The same study revealed that patients who died from COVID-19 had significantly higher D-dimer and fibrin degradation product (FDP) levels, a longer prothrombin time (PT), and a prolonged activated partial thromboplastin time (PTT) at presentation, as compared to those who survived [6]. Therefore, it is important to monitor hematological parameters during the disease course in hospitalized patients [7]. Coagulopathy complications reported in previous studies, such as an increase in D-dimer level, were witnessed in our patient who was consequently placed on low molecular weight heparin (LMWH).

There is almost a global consensus that anticoagulants have a pivotal role in treating COVID-19. A recent observational study that included more than 4000 patients in the United States revealed that early initiation of prophylactic anticoagulation within 24 hours of admission reduced mortality risk by 27% [8]. Most healthcare systems have established protocols for hospitalized patients with COVID-19 to receive pharmacologic thromboprophylaxis with LMWH or fondaparinux unless the risk of bleeding is higher than that of thrombosis. Different recommendations for prophylaxis and treatment of venous thromboembolism (VTE) have been recommended by several scientific organizations. The latest British National Institute for Health and Care Excellence (NICE) guidance suggests giving therapeutic doses of LMWH to hospitalized patients in medical wards who are expected to be admitted for more than three days, or any patient who needs O2 supplementation [9]. In the United States, the National Institute of Health (NIH) recommends prophylactic doses of anticoagulation for all hospitalized patients and reports no sufficient evidence for higher doses. It also recommends considering thromboembolic disease in any COVID-19 positive patient who rapidly deteriorated clinically [10].

Several studies have looked into the best dose recommendations for better clinical outcomes, with no conclusive answer yet. Some studies found that a full dose of LMWH reduced the need for life support level of care and improved clinical outcomes [11]. In critically ill patients, other studies have found that high doses of prophylaxis decreased the level of thrombosis without increasing the risk of bleeding [12]. On the other hand, there is evidence pointing in the other direction such as a study on more than 400 hospitalized patients reporting no mortality benefit for therapeutic doses, and increased complications [13].

Our case revealed that the decision to use therapeutic dose anticoagulation becomes much more challenging if patients refuse blood transfusion. Being knowledgeable of alternative medical interventions that can be used if patients needed a blood transfusion was crucial in this case. JW patients generally refuse whole blood and blood component transfusion. However, many will accept blood derivatives such as albumin, clotting factor concentrates, immunoglobulins, and cryoprecipitate. In addition, pharmacological interventions can be used as well, and are largely counted on for treating patients who refuse blood transfusion. Tranexamic acid, recombinant activated factor VII (rFVIIa), parenteral iron preparation, and erythropoiesis-stimulating agents (ESAs) are among accepted interventions by JW patients [14].

It is worth noting that medical approaches to help patients who refuse blood transfusion are based on the clinical context. In surgical cases, autologous blood transfusion techniques such as intraoperative and postoperative cell salvage are generally accepted by JW patients whereas preoperative autologous blood transfusion is not. Acute hypervolemic hemodilution is another example of tolerated solutions by JW patients that can be used in a surgical context [15]. Red blood substitutes such as hemoglobin-based oxygen carriers could have been a reasonable solution in this case, but they have not been approved yet for use in the United States [16]. Therefore, enoxaparin sodium was stopped and the patient's hemoglobin was closely monitored.

Our case uncovers the need for more research about bleeding events and possible complications of heparin and LMWH therapy in COVID-19 management. More knowledge about the side effects versus benefits of using high-dose anticoagulants will help clinicians around the world to risk assess their patients, and help to make a personalized decision in each case. A patient-centered approach balancing the risk of thromboembolism versus the risk of bleeding should be adopted when managing COVID-19.

This patient clearly presented a high risk for thrombotic disease as his D-dimer level was elevated more than five times than the normal limit. Therefore, the patient was placed on enoxaparin sodium, but he sadly developed retroperitoneal bleeding. 

## Conclusions

As we use anticoagulation, we run the risk of bleeding. However, anticoagulation is a critical step in the management of patients with severe COVID-19 to reduce the risk of both arterial and venous thrombosis. Our case is an example of COVID-19-associated coagulopathy, and the bleeding risk attendant on its management. This case was particularly complex regarding limited options of management as blood transfusion was refused. A personalized benefit-risk assessment is of paramount importance to safe practice in such situations. 

Clinicians should familiarize themselves with interventions to help JW patients if they develop bleeding as a complication of anticoagulation therapy. Early aggressive intervention, being aware of alternatives to blood transfusion available to the Jehovah's Witness population, and guideline-based use of anticoagulation can improve outcomes in such complicated cases.
